# Can Reproductive Life Plan-based counselling increase men’s fertility awareness?

**DOI:** 10.1080/03009734.2018.1541948

**Published:** 2018-12-13

**Authors:** Maja Bodin, Tanja Tydén, Lisa Käll, Margareta Larsson

**Affiliations:** a Department of Women’s and Children’s Health, Uppsala University, Uppsala, Sweden;; b Centre for Gender Research, Uppsala University, Uppsala, Sweden

**Keywords:** Counselling, fertility awareness, lifestyle, men, preconception care, reproduction, ClinicalTrials.gov Identifier, NCT02736214

## Abstract

**Background:** Many men have limited knowledge about reproductive health and fertility. The aim of the study was to evaluate if Reproductive Life Plan (RLP)-based counselling during a sexual health visit could increase men’s fertility awareness.

**Material and methods:** The study was a randomized controlled trial including 201 men aged 18–50 who visited either of two participating sexual health clinics in Sweden for sexually transmitted infection testing during 2014–2016. All men received standard care, and men in the intervention group (IG) also received oral and written RLP-based information about lifestyle and fertility. Awareness about fertility and lifestyle-related factors were the main outcomes, measured through a questionnaire before the intervention and through a telephone survey after three months. Impressions from the counselling were also assessed at follow-up.

**Results:** A majority (71%) of men wanted children in the future. General fertility awareness increased from a mean score of 4.6 to 5.5 out of 12 (*P* = 0.004) in the IG. The mean number of accurate lifestyle factors (that could affect fertility) mentioned increased from 3.6 to 4.4 (*P* < 0.001) in the IG. There were no improvements in the control group. Among the men in the IG, 76% had a positive experience of the counselling, and 77% had received new information.

**Conclusion:** The intervention managed to increase different aspects of men’s fertility awareness. In the future, the format for preconception care for men needs further development. Including men in preconception health policy guidelines and identifying suitable actors for care provision would be important first steps.

## Introduction

Lifestyle before conception may influence the reproductive performance of women and men, as well as the well-being of a future child. Thus, there is strong evidence that age, weight, and smoking can have an adverse effect on reproduction (1). It has also been suggested that factors such as diet, exercise, stress, alcohol, illicit drugs, radiation, pollution, and exposure to chemicals can have negative effects, although the evidence is less conclusive (1,2). Several studies have found that many people have limited awareness of factors that influence their reproductive health and fertility (3–8). Improving access to preconception care could be one way of addressing this matter. Since lifestyle-related factors are potentially modifiable, it has been recommended that people of child-bearing age should be counselled and advised about individual lifestyle factors in relation to reproductive goals, as a health preventive measure (9,10). Still, guidelines for preconception care are missing in several countries, and men are rarely targeted with preconception health information (11). The inattention to men’s reproductive health often implies that women get or take the blame for involuntary childlessness and foetal harm, while men take their fertility for granted or stay silent about their worries (12,13). Hence, there is a need to start talking to men about reproductive health and fertility.

### Reproductive life planning

To motivate individuals to reflect on personal reproductive goals and to outline a plan to achieve them, the Centre for Disease Prevention and Control in the USA has developed a tool named the Reproductive Life Plan (RLP) (14,15). The RLP tool provides a structured format for people to reflect about their desires to have or not to have children in the future. The RLP can be reviewed privately or in conversation with a health care provider (HCP) during preconception counselling. If discussed during counselling, the HCP has the opportunity to give the individual or couple information and support concerning fertility, reproductive health, and potentially modifiable risk factors. Hence, it has the potential to raise what is internationally defined as fertility awareness, i.e. ‘the understanding of reproduction, fecundity, fecundability, and related individual risk factors (e.g. advanced age, sexual health factors such as sexually transmitted infections, and lifestyle factors such as smoking, obesity) and non-individual risk factors (e.g. environmental and work place factors); including the awareness of societal and cultural factors affecting options to meet reproductive family planning, as well as family building needs’ (16).

Evaluations of RLP-based counselling with women have indicated positive results (17,18), although an RLP can also be perceived as challenging or less meaningful to those who, for example, do not have clear pregnancy intentions (19). For successful implementation of RLP-based counselling, it is also important to firmly anchor the new concept among health care providers and managers at the clinics concerned, and offer education about fertility and training in how to use the guidelines (20).

If, when, and how RLP-based counselling should be offered to men has been given relatively little attention, even though the importance of preconception care for men has been elevated in the past 10 years (9,21,22). At present, several European countries, including Sweden, lack specific guidelines regarding preconception health information to men (11). In Sweden, sexuality education in schools has been mandatory since 1955, but the quality and quantity vary (23). Sexual health care is offered to young men and women in youth clinics and to adult women in family planning clinics as well as from private and public gynaecologists. Men have more limited options. Some larger cities have sexual health clinics, and some hospitals have genitourinary medical clinics, specialized in sexually transmitted infections (STIs).

### Men’s health-seeking behaviour

Men are often described as less engaged than women in health-promoting behaviour, and the way men reject health promotion behaviour can be viewed as a way of dissociating from ill-health, as ill-health is associated with weakness and dependence, which are often regarded as feminine traits (24). However, men’s health and health behaviour must not be understood as static, rather as related to norms of gender, class, nationality, age, and living conditions (25). What is considered as appropriate masculine behaviour therefore varies with time and space, and is changeable. There has been an increased attention to men’s health and bodies in society and media in the last years (26), indicating some changes in societal norms around health. According to studies on men who are engaged in healthy practices, men justify their health interest with reference to action orientation, autonomy, responsibility, and other traditionally masculine traits, which enable them to keep their masculinity ‘intact’ (27,28). Whether or not men’s increased interest in health includes reproductive and preconception health has not been well explored.

Men tend to seek sexual and reproductive health care less often than women do, which makes them less available for counselling. Instead of attending health clinics, young men are more likely to seek health information online (29). There have been some attempts globally to increase men’s awareness of fertility and reproduction by online education websites (30) and information brochures (31,32). To our knowledge, no study has evaluated the effectiveness of face-to-face preconception counselling among men. Hence, the purpose of this study was to investigate if RLP-based counselling during sexual health visits could increase men’s awareness about reproductive health and fertility, and to evaluate the reception and acceptance of the counselling. This could provide indications about the possibilities of implementing preconception care for men in the Swedish context, and possible policy changes for the future.

## Material and methods

The study was designed as a randomized controlled trial. We decided to target men attending sexual health care because of their increased likelihood of having unplanned pregnancies and STIs that could affect fertility. The intervention took place at one larger STI and sexual health clinic in the capital of Sweden (Clinic A) and at one smaller clinic targeting men only, situated in a major university city (Clinic B). Clinic A had about 3000 male visitors per year and several midwives employed to take care of STI testing and sexual health counselling. Participants were recruited at drop-in hours. Clinic B was integrated in a larger health centre; it was open one day a week and was profiled towards men aged 20–29. Most visits at Clinic B were pre-booked, and eligible participants were approached in the waiting room. Inclusion criteria were ability to read and speak Swedish, and being a male 18–50 years of age. The power calculation was based on the results from a similar study with women (17), and more specifically on the increase in knowledge about reproduction, measured by four specific questions. In the study by Stern et al. (17), the intervention group increased their knowledge from 2 to 3.5 points out of 8. To be able to detect a percentage-like increase in the current study, at least 64 participants per group would be needed. Since the topic of the intervention was regarded as sensitive, a rather high dropout rate was expected, and the aim was therefore to recruit 100 men to each group.

**Figure 1.**
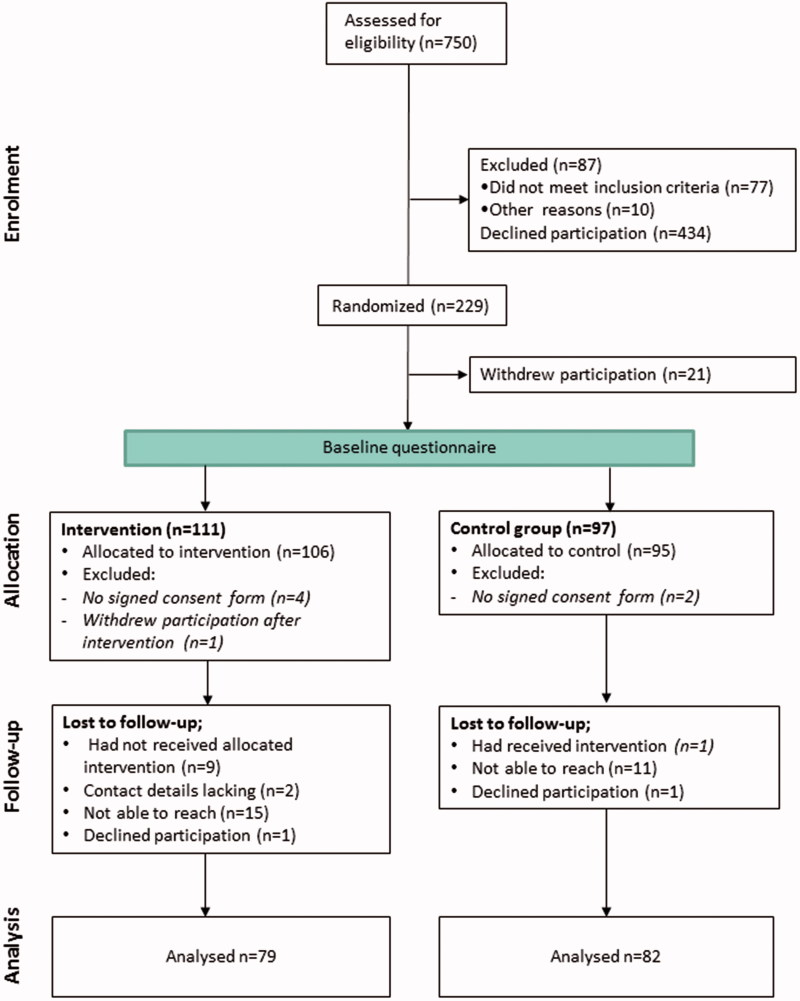
CONSORT flowchart.

A flowchart of the study procedure is presented in Figure 1. At least 750 men were approached for eligibility; 77 of these were excluded since they did not meet the inclusion criteria and 10 due to other circumstances (e.g. being psychologically unstable or under the influence of alcohol). Eligible clients were given oral and written study specific information. In total 434 men declined participation. Among the 229 who had accepted participation, 21 men withdrew after randomization, one withdrew after the intervention, and six men were excluded since they had not signed the consent form. Hence, 201 men remained in the baseline sample: 106 in the Intervention Group (IG) and 95 in the Control Group (CG). Among the 201 participants included in the baseline measurements, 40 (20%) were lost to follow-up since they did not answer the phone (*n* = 26), declined participation (*n* = 2), had not provided contact details (*n* = 2), or had been treated by the nurse-midwife as belonging to the opposite study group (*n* = 10).

### 

#### The intervention

The intervention, i.e. the RLP-based counselling, was carried out by nurse-midwives, the category of health care providers who, in Sweden, are usually responsible for sexual and reproductive health care to healthy young men and women. All participants received standard care (e.g. STI testing). During the intervention, the nurse-midwives used the RLP-tool and a list of fertility facts as guidelines for conversation, and checked off that all topics had been discussed. The session started with the question of whether or not the participating man planned to have any (more) children in his life. Based on the response, further questions were posed and the counselling continued by emphasizing relevant information on fertility and lifestyle recommendations to those who would like to become biological parents according to the predetermined checklist. The participants in the IG also received a brochure designed specifically for the intervention about male fertility and lifestyle.

#### Instrument

To assess men’s knowledge about fertility and related lifestyle factors, before and after the intervention, as well as their perceptions of the counselling, a questionnaire was used. The questionnaire included questions about background characteristics (including sexual and reproductive health history and child visions), six open-ended knowledge questions about reproduction, and two open-ended questions about lifestyle factors relevant to fertility.

The exact formulation of the questions was study-specific but based on The Swedish Fertility Awareness Questionnaire used in several previous studies (6,33). We used the same questions as in the above-mentioned study among women (17) but added questions about sperm and male infertility. The six knowledge questions were phrased as follows:

1. How long is the ovum viable for fertilization after ovulation?
2. How long does sperm usually survive in the uterus/fallopian tubes after intercourse?
3. How likely is it that a 25-year-old woman becomes pregnant if she has unprotected intercourse with a young man at the time of ovulation?
4. At what age is there a marked decline in a woman’s ability to become pregnant?
5. How often is involuntary childlessness among heterosexual couples caused by a male factor?
6. What are the average chances of having a child through IVF, after each attempt?

To investigate knowledge of fertility-related lifestyle factors, participants were asked to write as many factors as they were aware of that could affect male fertility. They were also asked about any lifestyle changes a man could make prior to a future pregnancy to improve the likelihood of conception and having a healthy child.

#### Procedure

Before the study began, the principal investigator (M.B.) and a senior investigator (M.L.) instructed the nurse-midwives about the RLP-tool and the study procedure to ensure equivalent implementation of the intervention. All nurse-midwives had previous training in andrology and experience from counselling men. Data collection began in October 2014 at Clinic A. Clinic B was involved in May 2015 because of a prolonged recruitment process. The goal to recruit 200 men was achieved by February 2016.

Potential participants were recruited by a receptionist or by a nurse-midwife in the waiting room. Eligible clients were given oral and written study-specific information. The invitation letter distributed to participants described the study as an investigation of a new model for reproductive health counselling. Men who accepted participation (*n* = 229) were randomized into two groups by picking up a sealed, colour-coded envelope from a box. Half of the envelopes were coded as IG and the other half as CG. All envelopes contained a letter of consent, a baseline questionnaire and an instruction letter. The participants completed the consent form and the baseline questionnaire in the waiting room. When the participant entered the consultation room, the nurse-midwife could determine the group allocation from the colour code on the envelope. During the counselling, the nurse-midwives ticked off the topics covered on the checklist. All checklists were returned when the study was completed and according to them all topics had been discussed, at least to some extent, with every man.

Three months after the visit, the principal investigator phoned the participants for a structured follow-up interview. Participants were asked to answer the same knowledge questions about reproduction and lifestyle factors as at baseline. Additionally, patients in the IG were asked about their experiences of the RLP-based counselling. Spontaneous comments were noted in the margin of the questionnaire and were considered as part of the study findings. After the interview, participants in the CG were offered the brochure that had been given to the IG.

#### Ethical consideration

The information about the study ensured that participation was voluntary and could be ended at any time without any stated reason. The Regional Ethical Review Board in Uppsala approved the study.

### Statistical analysis

Statistical analyses were performed using SPSS version 24 and SAV version 9.4. The statistical analysis concerns two main outcomes: 1) general fertility awareness; and 2) awareness of lifestyle factors that could affect men’s fertility. General fertility awareness was measured by the six knowledge questions mentioned above, which had open-ended answers. Each answer was transformed to a score (0–2 points). Scores were given according to a correction template developed from available literature and in discussion with experienced clinicians. The six scores were then computed to form a total score, ranging from 0 to 12 points. As for awareness of lifestyle factors, the factors considered accurate were summed up to a total number.

Although discrete, the total score for reproductive knowledge was treated as a continuous variable since the scores were normally distributed and a sensitivity test supported that parametric tests could be used. Independent *t* test and one-way ANOVA were used to compare means at baseline between subgroups (background characteristics). To measure if the intervention led to any improvement in knowledge and awareness, paired samples *t* test was used to analyse differences in means between baseline and follow-up within groups. Next, analysis of covariance was performed through a general linear model to evaluate whether the effect of the intervention was associated with other variables. The categorical variables included in the model were level of education, relationship status (stable romantic partner or not), being a father or non-father, and wanting children or not. Age was included as a continuous variable. The results are presented with two-sided *p* values, where *p* ≤ 0.05 is considered statistically significant.

Seventy-three men commented their answers during the structured interview at follow-up. Since most comments were very brief, no content analysis was performed. However, citations are used verbatim to illustrate the quantitative findings.

## Results

### Characteristics of participants

No statistically significant differences in characteristics were found at baseline between the IG and the CG (Table 1). Nor did the participants lost to follow-up differ from the other participants in terms of background characteristics. As described, many men had previously had an STI, and the most frequent type was chlamydia (75% of those affected). One out of three had been involved in at least one pregnancy, and a majority of these pregnancies had been terminated with induced abortion (Table 1).

**Table 1.** Characteristics at baseline of men in the intervention group (IG) and the control group (CG).

	IG (*n* = 106) (%)	CG (*n* = 95) (%)	Total (*n* = 201) (%)
Age			
Years; mean ± SD	28.5 ± 6.7	28.3 ± 5.6	28.4 ± 6.2
Level of education (highest completed)			
Primary school	3.8	3.2	3.5
Secondary school	50.0	49.4	49.7
University	46.2	47.4	46.8
Country of birth			
Sweden	85.7	84.2	85.0
Other European	3.8	9.5	6.5
Non-European	10.5	6.3	8.5
Type of relationship^a^			
Main partner	35.6	42.6	38.9
Regular sexual partner	18.3	14.9	16.7
Casual known	26.0	25.5	25.8
Casual unknown	14.4	6.4	10.6
No partner	22.1	20.2	21.2
Reproductive history			
Contraceptive method used at latest sexual encounter^a^			
None	25.5	24.5	25.5
Coitus interruptus/safe period	9.4	8.5	9.0
Condom	48.1	46.8	47.5
IUD	12.3	12.8	12.5
Birth control pill/NuvaRing/p-rod	29.2	27.7	28.5
Don’t know	2.8	2.1	2.5
Never had sex	0	1.1	0.5
History of sexually transmitted infection			
Yes	44.3	33.7	39.3
No	55.7	65.3	60.2
Don’t know	0	1.1	0.5
History of conception			
Yes, several times	14.3	9.5	12.0
Yes, once	24.8	17.9	21.5
No	61.0	72.6	66.5
Difficulties conceiving	1.9	0	1.0
History of abortion	25.7	21.1	23.5
History of miscarriage	4.7	5.3	5.0
History of childbirth	13.2	7.4	10.4

a Several options could be chosen.

A total of 71% wanted to have children in the future. At follow-up, a greater share of men in the IG (76%) wanted to have children, than at baseline (58%). According to men who were not yet fathers, the most preferable age to have the first child was at 32 and the last child at 38. These figures had not changed at follow-up.

### General fertility awareness

At baseline, the mean knowledge score was 4.6 ± 1.9 out of 12 for the whole sample (*n* = 201) (Table 2). There was no difference in knowledge between the IG and the CG. Fathers (*n* = 21) had a higher mean score than non-fathers (5.4 ± 1.7 and 4.5 ± 1.9, respectively, *p* = 0.046). Men with secondary education had lower knowledge than men with university education (mean score 4.3 ± 2.0 and 5.0 ± 1.9, respectively, *p* = 0.043).

**Table 2.** Distribution of knowledge scores and awareness of lifestyle factors in the IG and the CG, at baseline and follow-up.

		IG			CG		
		Baseline (*n* = 104)	Follow-up (*n* = 79)	*P* value	Baseline (*n* = 94)	Follow-up (*n* = 81)	*P* value
1. Knowledge score	Mean	4.6	5.5	0.004	4.6	4.7	ns
	Median	5	6		5	4	
	Min–max	0–10	0–11		1–9	0–10	
		(*n* = 106)	(*n* = 79)		(*n* = 95)	(*n* = 82)	
2. Lifestyle factors	Mean	3.6	4.4	<0.001	3.3	3.5	ns
	Median	3	5		3	3	
	Min–max	0–7	0–8		0–9	0–7	

ns = non-significant.

At follow-up, men in the IG had increased their mean knowledge score from 4.6 ± 2.1 to 5.5 ± 2.2, whereas there was no improvement in the control group. The linear model confirmed that the intervention had had a positive effect on reproductive knowledge (*p* = 0.012), and that the effect was not associated with any of the possible confounders in the model.

### Awareness of lifestyle factors

**Figure 2.**
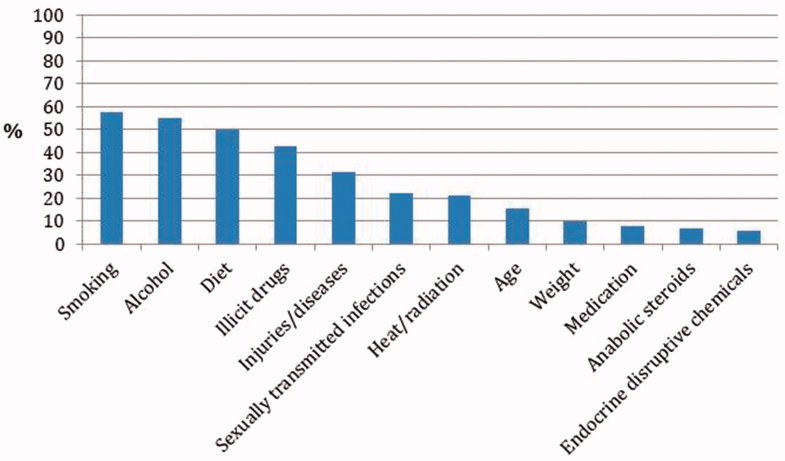
Percentage of participants aware of specific lifestyle factors that can affect male fertility, baseline measurement (*n* = 201).

At baseline, the total mean number of accurate lifestyle factors mentioned was 3.4 ± 1.8. The most commonly mentioned factors were tobacco use (mentioned by 58%), alcohol (55%), and unhealthy diet (50%) (Figure 2). There was low awareness concerning the possible impact of STIs, age, weight, and endocrine-disrupting chemicals. Beyond the factors discussed in the intervention, several other factors were mentioned such as wearing tight underwear, bicycling, mental ill-health, stress, and lack of sleep. These factors were not considered accurate and thus not included in the statistical analysis.

At follow-up, men in the IG had increased their mean from 3.6 ± 1.9 to 4.4 ± 1.6 factors. There was no improvement in the CG. The linear model confirmed that the intervention had had a positive effect on awareness of lifestyle factors (*p* < 0.001) and that the effect was not associated to any of the other variables in the model.

### Men’s experiences of the intervention

Most men had a positive experience of being asked about their RLP and received new information (Table 3). Only two men had a negative view of the counselling, which they related to the counsellor’s attitude towards their sexual behaviour. About one out of four men in the IG had never previously thought about the matters discussed and equally many stated that the counselling had raised new thoughts about fertility. One man believed that the reason that he had never talked about fertility before was a lack of interest in the topic and a fear of talking about things he knew little about. In the IG, 26% had talked about fertility with someone they knew after receiving the intervention. As an example, one man said he had dared to ask his girlfriend about her menstrual period after having had the RLP conversation. In the CG, 44% had talked about fertility with someone they knew after having participated in the study.

**Table 3.** Assessment of the RLP-based counselling by 78 men in the IG.

Question	*n* (%)
Had previously thought about the matters of fertility discussed during the intervention	
Not at all	21 (27)
Fairly little	27 (35)
Neither little nor much	18 (23)
Fairly much	10 (13)
Much	2 (2)
Information during counselling perceived as new	
Nothing	2 (3)
Fairly little	5 (6)
Neither little nor much	11 (14)
Fairly much	48 (62)
Much	12 (15)
Experience of being asked about RLP by a nurse-midwife	
Very negative	1 (1)
Fairly negative	1 (1)
Neither negative nor positive	17 (22)
Fairly positive	23 (30)
Very positive	36 (46)
The consultation raised new thoughts about fertility	
To a very small extent	14 (18)
To a fairly small extent	23 (29)
Neither small nor large extent	19 (24)
To a fairly large extent	18 (23)
To a very large extent	4 (5)
The consultation led to a search for more information about fertility	
No	53 (68)
Yes	25 (32)
Likelihood of consulting a nurse-midwife if questions about fertility arise	
Very unlikely	8 (10)
Fairly unlikely	8 (10)
Neither likely nor unlikely	11 (14)
Fairly likely	25 (33)
Very likely	25 (33)
Perceived importance of educating young men about fertility and factors that can affect a healthy pregnancy	
Very unimportant	1 (1)
Fairly unimportant	2 (3)
Neither unimportant nor important	1 (1)
Fairly important	23 (30)
Very important	51 (65)

A majority (66%) of the participants in the IG would turn to a nurse-midwife again if they wanted more information about fertility, although several commented that they would first ‘google’ or that they would not search for more information on fertility until planning to conceive a child (Table 3). Some suggested that a homepage or mobile phone application about fertility would be useful. Three out of four responded that they would be likely to make a preconception lifestyle adjustment in the future if planning for pregnancy. However, several men commented that they would not change anything since they already lived a sufficiently healthy life. Others stated that they would not make any adjustments until experiencing difficulties conceiving. Almost all men, 95%, in the IG agreed that it is important to educate young men about the matters discussed during the intervention (Table 3). One man said he was very surprised by how little he actually knew. Another man was upset about how skewed knowledge on pregnancy and fertility is today; that men only hear that they should beware of pregnancy. Frustration about not having access to sexual and reproductive care was also expressed by a few. However, it was also stated that preconception counselling should only be given to men ‘in the danger zone’, and not as a routine during a sexual health visit. Talking about RLP as a routine could be negative if the man was there for another purpose. ‘If you have other problems you will not be comfortable with the questions’ was an opinion expressed by several men. In their opinion, it would therefore be better if the man himself took the initiative to talk about his reproductive health.

## Discussion

This study has shown that brief RLP-based counselling during a sexual health visit can raise men’s awareness about reproductive health and fertility. We cannot draw any conclusions concerning effects on behaviour, but we know that the counselling was positively received by most men and generated some new thoughts and conversations about fertility. Even in the control group, several men began to talk to friends or partners about fertility and reproduction merely after having completed the baseline questionnaire. Curiosity was raised, which indicates that just introducing the topic may make men mindful about their procreative intentions. Hence, giving men an opportunity to talk about their goals could make them reflect on their sexual and procreative identities and responsibilities within a larger context (34). However, the subject was delicate to some and clearly not a self-evident topic of conversation.

### Timing and perceived relevance

The intervention had a small but measurable effect on participants’ fertility awareness. Why the awareness did not increase even more could be related to timeliness; several men expressed that this type of information is irrelevant until it is time to conceive. Not seeing a pregnancy in the near future or being uncertain about long-term pregnancy goals has previously been identified as a pitfall with reproductive life planning. This could partly also explain the difficulties with recruiting participants to the study. According to the staff involved in the study, many men were clearly taken by surprise when asked to talk about their reproductive health. However, one could also argue that this is related to contemporary expectations on gender; men are not expected to talk about this topic, while women clearly are. In a similar study with women at a student health centre, only 25 out of 338 eligible women declined participation (17). This is a striking contrast to our study. Refusing to participate in the study could be understood as unwillingness or unfamiliarity associated with both ill-health and the feminized area of reproduction.

Time was also relevant in the sense that many men attended the clinic at drop-in hours and expected to have a quick STI test. One of the most common reasons not to participate was ‘no time’; participating in a randomized controlled trial (RCT) was obviously seen as time consuming. Another reason not to participate could be that leaving identification data to researchers for follow-up is a sensitive matter. From the comments at follow-up, we can clearly see that both sexual health and fertility are sensitive topics.

### Mode of communication

Our study confirms that many young men prefer finding information about sexual health online (29). Several apps about fertility and reproductive functions are today available, but almost all are directed to women (35). Developing a fertility app for men could be an option to increase men’s knowledge and engagement in reproductive health. A pitfall of apps is the loss of personal encounters between patient and caregiver, and there are potential negative consequences of constant monitoring of health and putting responsibility for health on individuals that need to be taken into account (35). As for the internet, there is today an extensive mass of information provided by unclear sources on the web, which can make it difficult for a reader to interpret what information to trust. In this case, governmental organizations ought to take the responsibility to provide reliable, inclusive, and comprehensible information, with a clear sender, as has been done for example in the USA and in Australia (36,37). In Sweden, a new mobile-friendly website with evidence-based information on fertility and lifestyle has recently been developed by our research group (www.reproduktivlivsplan.se) (38). A possible improvement of our trial could have been to offer participants access to an interactive app or homepage instead of the brochure, since most young men in Sweden today use smart phones. This could have generated more curiosity and maybe increased knowledge. An app is also more discreet than a brochure and a good solution for those who found the topic too sensitive to talk about.

### Strengths and limitations

This is to our knowledge the first RCT that evaluates face-to-face preconception counselling with men. We managed to raise men’s fertility awareness, and we have shown that several men would like to have better access to preconception counselling and information. This is an important finding for policy implications.

However, a factor that limits us from generalizing the results is the study sample. By showing up at the clinic in the first place, the participants already demonstrated a level of health awareness and health-seeking behaviour. These sample features, as well as the high frequency of previous STIs and abortions, should be kept in mind when drawing conclusions about men in general. On the other hand, the sample represents a group of men that could clearly benefit from receiving information about fertility and lifestyle, which makes the study even more relevant. As the results showed, few men were aware that STIs could impact fertility.

The interest in participating was lower than expected. To be able to finalize the study within an acceptable time frame, a second clinic was approached when the study had been ongoing for half a year. Clinic B was located in a different city but had a similar clientele. The mean age was lower at Clinic B, and age was thus adjusted for in the statistical analyses. The goal to include 200 men was not achieved until 17 months after the initiation of the study, which could imply societal changes over time. However, to our knowledge, no societal initiatives or educational efforts that could have influenced men’s general knowledge about reproduction took place during this time period. What was different at Clinic B was that the visits often were pre-booked, and the midwives there did not to the same extent experience the lack of interest from men to participate. This suggests that the time at hand was a component that affected both the recruitment process and the success of the intervention. Hence, this ‘limitation’ contributed to the study with a valuable insight.

Finally, this study cannot be said to fully measure men’s fertility awareness (16). Even though we have tried to attend to different aspects included in the concept of fertility awareness, we believe that more questions and qualitative methods are needed to give a more comprehensive picture of how men think and what men know about reproductive health and fertility.

### Implications for future practice and research

As we see it, there are several challenges ahead in increasing men’s fertility awareness. Firstly, how can we make reproductive health feel relevant to them before they start having trouble conceiving? Secondly, who should be responsible for delivering preconception care to men? In Sweden, nurse-midwives are often responsible for delivering sexual and reproductive health care, but male health issues are not included in their formal education. As suggested by some participants, information about preconception health could also be given during sexual education, which means that school nurses, teachers, and other sexual health educators need to become involved and receive more training. As concluded in a previous study, a key factor for successful implementation is to have all the intended professionals on board and motivated to implement the new concept (20). These questions need further thought before scaling up preconception health care for men.

## Supplementary Material

Supplemental Material
